# Deriving an overall appearance domain score by applying bifactor IRT analysis to the BODY-Q appearance scales

**DOI:** 10.1007/s11136-019-02366-8

**Published:** 2019-11-22

**Authors:** Daan Geerards, Lisa van den Berg, Andrea L. Pusic, Maarten M. Hoogbergen, Anne F. Klassen, René R. W. J. van der Hulst, Chris J. Sidey-Gibbons

**Affiliations:** 1 Patient-Reported Outcomes, Value & Experience (PROVE) Center, Department of Surgery, Brigham and Women’s Hospital, 75 Francis St, Boston, MA 02115 USA; 2 Department of Surgery, Harvard Medical School, Boston, MA USA; 3 Department of Plastic and Reconstructive Surgery, Catharina Ziekenhuis, Eindhoven, The Netherlands; 4 Department of Pediatrics, McMaster University, Hamilton, ON Canada; 5 Department of Plastic and Reconstructive Surgery, Maastricht University Medical Center, Maastricht, The Netherlands; 6 Department of Symptom Research, University of Texas MD Anderson Cancer Center, Houston, TX USA

**Keywords:** Patient-reported outcome measures, Psychometrics, Bifactor, Item response theory, BODY-Q, Massive weight loss, Obesity, Body contouring, Appearance

## Abstract

**Purpose:**

With the BODY-Q, one can assess outcomes, such as satisfaction with appearance, in weight loss and body contouring patients using multiple scales. All scales can be used independently in any given combination or order. Currently, the BODY-Q cannot provide overall appearance scores across scales that measure a similar super-ordinate construct (i.e., overall appearance), which could improve the scales’ usefulness as a benchmarking tool and improve the comprehensibility of patient feedback. We explored the possibility of establishing overall appearance scores, by applying a bifactor model to the BODY-Q appearance scales.

**Methods:**

In a bifactor model, questionnaire items load onto both a primary specific factors and a general factor, such as satisfaction with appearance. The international BODY-Q validation patient sample (*n *= 734) was used to fit a bifactor model to the *appearance* domain. Factor loadings, fit indices, and correlation between bifactor *appearance* domain and *satisfaction with body* scale were assessed.

**Results:**

All items loaded on the general factor of their corresponding domain. In the *appearance* domain, all items demonstrated adequate item fit to the model. All scales had satisfactory fit to the bifactor model (RMSEA 0.045, CFI 0.969, and TLI 0.964). The correlation between the *appearance* domain summary scores and *satisfaction with body* scale scores was found to be 0.77.

**Discussion:**

We successfully applied a bifactor model to BODY-Q data with good item and model fit indices. With this method, we were able to produce reliable overall appearance scores which may improve the interpretability of the BODY-Q while increasing flexibility.

## Background

The BODY-Q is a patient-reported outcome measure (PROM) designed to assess outcomes of people who undergo weight loss and/or body contouring. The BODY-Q can be used over an entire trajectory from obesity through to weight loss and subsequent body contouring surgery. The original BODY-Q framework consisted of 18 independently functioning scales (i.e., subdomains) in three different top-level domains (referred to as overall appearance scores in bifactor literature): *appearance* (7 scales), *health*-*related quality of life* (*HR*-*QoL*) (5 scales), and *experience of care* (4 scales) [1]. Additional scales (i.e., *appearance of chest, nipples and stretch marks*, *appearance*-*related distress*, and *expectations*) have been developed and published [2–4]. The scales contain 4 to 10 items, all scored on a Likert scale from 1 (e.g., ‘Definitely disagree’ or ‘Very dissatisfied’) to 4 (e.g., ‘Definitely agree’ or ‘Very satisfied’). Raw scores are converted into scores ranging from 0 (worst) to 100 (best) [1]. The BODY-Q questionnaire is currently being administered in both paper-based and Web-based form in multiple countries. Recently, computerized adaptive testing (CAT) of the BODY-Q was developed, which can reduce the number of items that a patient would need to complete to obtain a reliable score for each BODY-Q scale [5].

Systematic review evidence suggests that the BODY-Q is a valid and reliable tool for measuring outcomes following weight loss and body contouring surgeries [6]. One of the features of the BODY-Q is the set of appearance scales that measure satisfaction with the body overall and for specific areas (upper arms, abdomen, back, buttocks, inner thighs, and hips and outer thighs). These scales were designed specifically for obese and massive weight loss patients.

However, there are some situations whereby overall appearance scores for body appearance could provide several benefits. Firstly, for example, an item about satisfaction with abdomen may contain not only information about how a patient feels about his/her abdomen but may also contain information about overall appearance. This latent information is not utilized in current unidimensional measurement models (i.e., the partial credit Rasch model). Secondly, individual scale scores may become more accessible to interpret if separate appearance scales scores can be related to an overall appearance score. Thirdly, providing feedback to patients and physicians is desirable in outcome assessment and is made less complicated by providing a few summary scores instead of up to 7 separate scale scores. Lastly, benchmarking results for health care insurance, clinics, clinicians, or even individual patients might become more straightforward with overall domain scores instead of up to 7 different scales scores.

Earlier studies have made use of a bifactor model in outcome assessment, especially in mental health and quality of life research [7–14]. To our knowledge, only Kleif et al. applied a bifactor model to a surgical population [15]. An analysis using the bifactor model may have the potential to establish an overall domain score, potentially resulting in the aforementioned advantages. This study explores the feasibility of producing summary scores of the BODY-Q *appearance* domain through regular scale administration by applying a bifactor model to the BODY-Q.

## Methods

### Patient sample

The data sample for the bifactor analysis consisted of 734 patients (403 weight loss patients and 331 body contouring patients) from different practices in the United States (185 patients), Canada (412 patients), and the United Kingdom (137 patients). Patient demographics and characteristics are available in literature elsewhere [1].

### Bifactor model

Bifactor analysis was first described by Holzinger and Swineford in 1937 and extended to a confirmatory multidimensional Item Response Theory (IRT) model by Gibbons and colleagues [13, 16, 17]. In a bifactor model, which is a hierarchical model, there is a two-level structure. All items are assumed to load on both a primary or overall appearance score (e.g., *satisfaction with appearance*) and a secondary or lower order dimension (e.g., *satisfaction with abdomen*) [18].

Items within a scale (e.g., *satisfaction with abdomen*) can have a high correlation, compared to items between scales (e.g., *satisfaction with abdomen* vs. *satisfaction with outer hips*). When this is the case, there are as many dimensions as there are scales (i.e., subdomains), which is a violation of unidimensional IRT. This violation could be dealt with by using a bifactor IRT model [19]. In the same approach as described, the bifactor model might be applicable to a BODY-Q *appearance* domain.

### Domains and scales

For the *appearance* domain, the *skin* and *scar* scales were excluded from the analysis as they are only applicable to some patients at some timepoints, *skin* for patients after massive weight loss with excess skin, and *scar* for patients after body contouring surgery [1]. All seven remaining scales were included in the analysis: *satisfaction with body, abdomen, upper arms, back, buttocks, hips and outer thighs*, and *inner thighs*.

### Analysis

Analysis was performed in R (version 3.4.3). The mirt package was used to estimate the bifactor models including multidimensional IRT parameters [20, 21]. Item fit values were derived by using the ‘itemfit’ function with item type set to graded response model. Factor loading values per item were collected with the ‘bfactor’ function, where each scale resembled a separate factor. Item parameters were derived with the ‘coef’ function within the mirt package. Patients undergoing surgery for cosmetic reasons only completed the scales related to their procedures (e.g., arms scale for brachioplasty patients and/or patients with excess skin on upper arms), whereas weight loss patients completed all appearance scales. Furthermore, respondents were not obliged to complete every item within a scale. Due to the nature of the mirt package, it was necessary to impute missing data (23%) in order to derive model fit statistics. Plausible values for missing data were therefore imputed using a 2PL graded response model for each of the separate subscales prior to assessment [21].

### Outcomes

Outcomes assessed were factor loadings (FL) of the scales within the *appearance* domain, Chi square statistics, root mean square error of approximation (RMSEA) [22], Tucker-Lewis Index (TLI) [23], and comparative fit index (CFI) [24]. Factor loadings can be described as a standardized regression coefficient. These values indicate how strongly an observed variable (i.e., an item) relates to one or more underlying latent factors (i.e., scale or domain score) and are considered as strongly related if a value is 0.4 or higher [25]. The Chi square value illustrates if an observed variable score corresponds to the expected variable score. A non-significant Chi square value (*p *> 0.01) indicates that the item fits; however, Chi square statistics are more prone to bias in large samples, such as ours [26]. Other fit indices, such as RMSEA, TLI, and CFI, take sample size into account [27]. Based on research using structural equation modeling (SEM), TLI and CFI values above 0.90 indicate adequate fit. Similarly, for RMSEA, a value below 0.05 represents a good fit, and a value higher than 0.10 represents a poor fit. [22, 27, 28].

We evaluated the usefulness of the overall appearance score with the estimated common variance (ECV) statistic. The ECV statistic is a useful indication of extent to which the general factor explains the variance in scores [14]. The statistic ranges from 0 to 1 where 1 is perfectly unidimensional. Though few studies have evaluated the validity of different thresholds for the ECV statistic, a value of .90 or greater than .90 could be considered essentially unidimensional, and below .70 sufficiently multidimensional to fit the data to a multidimensional IRT model [29].

We assessed the correlation between the *appearance* bifactor domain scores, with the *satisfaction with body* scale excluded, and original *satisfaction with body* scale scores. We also determined the correlation between all 7 subscales (Table 1).

**Table 1:** Satisfaction with Body Scale. Item descriptions are not intended for replication. Please visit the Q Portfolio website for full item wording

Item content*	Very dissatisfied	Somewhat dissatisfied	Somewhat satisfied	Very satisfied
1. …looks when dressed	1	2	3	4
2. …how clothes fit	1	2	3	4
3. …size	1	2	3	4
4. …Shape	1	2	3	4
5. …looks in photos	1	2	3	4
6. …looks from behind	1	2	3	4
7. …Looks from the side	1	2	3	4
8. …Looks in summer clothes	1	2	3	4
9. …Looks in a swimsuit	1	2	3	4
10. …Look in a mirror unclothed	1	2	3	4

## Results

All factor loadings for the corresponding items can be seen in Table 2. It was found that all items (*n *= 42) had substantial loadings onto both the primary and overall appearance factors (FL > 0.40, FL > 0.69, respectively), indicating that all BODY-Q items represent valuable components of the primary or overall appearance factor (i.e., that these items were adequately related to overall *appearance* satisfaction).

**Table 2:** Appearance items and factor loadings (^2^ = Chi square, df = degrees of freedom)

Scale	Items	Factor loadings								Item fit for primary factor		
		Primary factor	Body	Abdomen	Upper arms	Back	Buttocks	Hips and outer thigh	Inner thighs	^2^	df	p^2^
Body	1. Looks when dressed	0.824	0.446							89. 086	86	0.388
	2. How clothes fit	0.764	0.524							86.473	95	0.722
	3. Size	0.850	0.415							86.129	84	0.415
	4. Shape	0.781	0.467							95.226	89	0.306
	5. Looks in photos	0.804	0.479							117.523	94	0.051
	6. Looks from the behind	0.883	0.303							60.422	77	0.918
	7. Looks from the side	0.843	0.288							70.043	73	0.576
	8. Looks in summer clothes	0.904	0.195							66.743	66	0.451
	9. Looks in a swimsuit	0.926	0.105							58.939	62	0.587
	10. Looks in mirror unclothed	0.930	0.083							54.827	48	0.232
Abdomen	1. How clothes fit	0.857		0.459						72.373	77	0.628
	2. Size	0.862		0.453						67.326	71	0.602
	3. Looks from the side	0.878		0.373						37.724	54	0.955
	4. Shape	0.847		0.454						79.861	87	0.694
	5. Looks in a swimsuit	0.862		0.457						63.472	69	0.665
	6. How toned	0.896		0.354						65.640	64	0.420
	7. Looks when naked	0.897		0.353						57.039	48	0.174
Upper arms	1. Size	0.760			0.465					84.389	90	0.647
	2. How smooth	0.766			0.503					88.027	92	0.598
	3. Shape	0.655			0.560					130.316	100	0.022
	4. How skin looks	0.682			0.521					95.507	102	0.662
	5. How toned	0.743			0.521					104.574	89	0.124
	6. Look when lifted up	0.762			0.508					104.286	89	0.128
	7. Look when not covered	0.706			0.537					106.976	100	0.298
Back	1. How smooth	0.846				0.399				71.771	66	0.293
	2. Looks from different angles	0.829				0.447				101.008	76	0.029
	3. How toned	0.848				0.448				56.724	66	0.785
	4. Looks when naked	0.845				0.418				106.297	81	0.031
Buttocks	1. Size	0.847					0.378			80.269	81	0.502
	2. Look from the side	0.847					0.415			75.202	81	0.661
	3. Shape	0.816					0.427			96.342	89	0.279
	4. How smooth	0.834					0.390			88.265	87	0.442
	5. How skin looks	0.847					0.379			72.304	81	0.744
Hips and outer thighs	1. Size	0.887						0.382		67.642	72	0.624
	2. Shape	0.880						0.391		57.154	71	0.883
	3. How skin looks	0.873						0.384		66.725	72	0.654
	4. How smooth	0.868						0.362		60.471	74	0.871
	5. Look from behind	0.878						0.361		74.897	70	0.323
Inner thighs	1. How smooth	0.769							0.517	83.268	86	0.563
	2. How skin looks	0.765							0.535	86.415	84	0.407
	3. How toned	0.801							0.453	78.241	75	0.376
	4. Look when naked	0.786							0.478	86.944	76	0.184

The highest loading item was *“How your body looks in the mirror unclothed?”* (FL = 0.930). The lowest loading item was *“How satisfied are you with the shape of your upper arms?”* (FL = 0.655).

Without modification, all 42 items in the *appearance* domain demonstrated an adequate fit to the model based on a *p* > 0.01 criterion. Model fit was shown to be good with an RMSEA of 0.045 (90% CI 0.043–0.048). In addition, CFI and TLI are above recommended values for adequate fit (CFI = 0.969, TLI = 0.964). The ECV value for the combined appearance scale was − .85, suggesting that the bifactor model was appropriate to use in this case.

Multidimensional IRT parameters are displayed in Table 3.

**Table 3:** Appearance item parameters

Scale	Items	Discrimination parameters								Item intercepts		
		*a1*	*a2*	*a3*	*a4*	*a5*	*a6*	*a7*	*a8*	*d1*	*d2*	*d3*
Body	1. Looks when dressed	4.004	2.168							5.457	2.004	− 3.605
	2. How clothes fit	3.493	2.350							3.596	0.675	− 4.415
	3. Size	4.374	2.156							4.001	0.383	− 5.162
	4. Shape	3.191	1.907							4.010	0.853	− 3.369
	5. Looks in photos	3.870	2.321							2.857	− 0.136	− 5.053
	6. Looks from the behind	4.206	1.436							2.411	− 0.981	− 4.970
	7. Looks from the side	3.085	1.048							2.415	− 0.380	− 4.265
	8. Looks in summer clothes	4.052	0.900							1.911	− 1.141	− 5.846
	9. Looks in a swimsuit	4.268	0.500							0.286	− 2.494	− 7.076
	10. Looks in mirror unclothed	4.448	0.365							0.005	− 3.069	− 7.561
Abdomen	1. How clothes fit	2.679		1.595						2.617	− 0.284	− 3.542
	2. Size	3.148		2.077						2.701	− 0.606	− 4.755
	3. Looks from the side	2.263		2.007						1.647	− 1.334	− 4.552
	4. Shape	2.306		1.647						2.203	− 0.411	− 3.878
	5. Looks in a swimsuit	2.925		1.978						1.317	− 1.913	− 5.168
	6. How toned	3.203		2.135						0.768	− 2.333	− 5.914
	7. Looks when naked	3.056		2.179						0.597	− 2.287	− 5.664
Upper arms	1. Size	6.248			3.344					2.224	− 2.085	− 7.163
	2. How smooth	6.154			3.323					1.776	− 2.615	− 7.387
	3. Shape	5.000			2.186					− 0.017	− 3.295	− 7.214
	4. How skin looks	5.056			2.696					3.022	− 0.802	− 5.704
	5. How toned	6.749			3.488					2.031	− 2.477	− 8.073
	6. Looks when lifted up	5.775			2.345					0.408	− 3.042	− 7.745
	7. Looks when not covered	5.667			2.283					− 0.627	− 4.376	− 7.940
Back	1. How smooth	4.382				2.073				4.261	− 0.547	− 4.802
	2. Looks from different angles	4.368				2.342				4.822	0.319	− 4.833
	3. How toned	5.761				2.865				6.161	0.076	− 6.387
	4. Looks when naked	4.990				2.369				3.661	− 0.250	− 5.691
Buttocks	1. Size	3.982					1.811			3.619	0.021	− 4.487
	2. Look from the side	3.797					1.889			2.979	− 0.434	− 5.253
	3. Shape	3.240					1.651			2.280	− 0.877	− 4.932
	4. How smooth	3.337					1.592			2.365	− 0.828	− 4.977
	5. How skin looks	4.052					1.783			3.019	0.079	− 5.172
Hips and outer thighs	1. Size	3.478						2.370		0.553	− 2.782	− 6.195
	2. Shape	3.941						2.853		0.693	− 3.363	− 7.421
	3. How skin looks	3.493						1.952		0.105	− 3.230	− 6.488
	4. How smooth	3.530						2.097		− 0.099	− 3.251	− 7.118
	5. Looks from the behind	5.675							2.448	4.428	0.026	− 6.562
Inner thighs	1. How smooth	6.107							2.696	4.652	− 0.114	− 6.909
	2. How skin looks	4.709							2.087	3.385	− 0.488	− 5.660
	3. How toned	4.865							2.119	3.425	− 0.724	− 6.199
	4. Looks when naked	5.662							2.315	3.587	− 0.760	− 7.312

Correlation between *appearance* domain scores and *body* scale scores was found to be 0.77. Correlation between all subscales was high with values ranging between 0.63 and 0.83 as can be seen in Table 4.

**Table 4:** Subscale correlations (Pearson correlation coefficient)

Scale	Body	Abdomen	Upper arms	Back	Buttocks	Hips and outer thighs	Inner thighs
Body		0.83	0.65	0.78	0.76	0.79	0.64
Abdomen	0.83		0.64	0.74	0.72	0.74	0.60
Upper arms	0.65	0.64		0.68	0.67	0.68	0.67
Back	0.78	0.74	0.68		0.74	0.77	0.63
Buttocks	0.76	0.72	0.67	0.74		0.81	0.68
Hips and outer thighs	0.79	0.74	0.68	0.77	0.81		0.72
Inner thighs	0.64	0.60	0.67	0.63	0.68	0.72	

## Discussion

In this study, a bifactor model was applied to the BODY-Q. It was shown that this model is satisfactory for the BODY-Q *appearance* domain, with good item and model fit. Furthermore, the feasibility to produce overall appearance score from regular items with the bifactor theory was demonstrated. Correlation between subscales was found to be high between all scales, which further justifies a bifactor model.

This study has several strengths. Firstly, the BODY-Q sample was international and large, which was beneficial for the analysis. Also, the sample contained both weight loss and body contouring patients, which makes this study applicable to both patient groups. Secondly, the bifactor model makes use of latent and otherwise unused information in already existing items. Thirdly, with this method, a new extra score is derived from regular item administration while the original BODY-Q scale scoring is not altered in any way.

Though we analyzed data from multiple countries, which have previously been shown to be invariant across cultures in unidimensional Rasch analyses, we did not employ a multigroup bifactor analysis and thus cannot comment on any potential invariance between cultures for the overall appearance factor. [1, 30] Further research is recommended both to confirm the cross-cultural suitability of the overall appearance factor as well as the general stability of the item calibration across a larger sample of patients.

A straightforward example of the use of a bifactor model in health assessment is depression. Depression could be described as a single construct, but actually consists of different components, such as agitation, suicidal thoughts, sleep disturbances, and anxiety. With this in mind, depression could also be seen as a hierarchical construct, where each separate component measures not only its own construct but also a general factor (i.e., severity of depression). Another example is intelligence, which consists of different components, such as logic, reasoning, planning, and problem-solving [14, 18, 19].

The new scores could be useful for different purposes, such as benchmarking, or for enhanced interpretation of PROM scores. The granular insight given by individual scales are useful tools for assessing prospective trials of specific single-site procedures, but the scores on an individual scale might not fully reflect the impact of extreme weight loss on patients. We envision that the overall score for the appearance scale may more accurately reflect the incremental improvement in satisfaction with global appearance which occurs with single-site surgeries. This overall appearance order measure may therefore also be useful for comparing different single-site operations in terms of their overall impact on bodily satisfaction.

The bifactor model could also be useful when providing feedback, where it would be easier to discuss a few summary scores instead of more than a dozen different scores. Fourthly, as in the original BODY-Q, all possible combinations of any of the scales can still be used according to the desire of the physician or researcher. Furthermore, multiple fit indices were analyzed, with most fit indices values being adequate or good. Lastly, a high correlation was found between the bifactor overall order *appearance* score and the regular *satisfaction with body* scale scores. This high correlation supports the rationale that confirms that the *satisfaction with body* scale is a satisfactory measure of overall body satisfaction, but also shows that the overall order *appearance* domain could be used as a surrogate for the *satisfaction with body* scale.

Our study does contain some notable limitations. Firstly, it can be difficult to accurately assess model fit and interpretability for the bifactor model, which is known to be at risk of overfitting. However recent research has shown that overfitting is not always the case but utilizing traditional information theoretic criteria, such as the Akaike information criteria (AIC) or Bayesian information criterion (BIC) [31–33]. Unfortunately, we were unable to calculate these statistics for our model. Additional uncertainly is brought about by the necessity on relying on item fit statistics which are suitable for SEM analysis and, despite popular usage, have not to our knowledge been confirmed as suitable for IRT analyses. Secondly, we had to rely on imputation to derive model fit statistics, due to missing data within the sample and nuances of the statistical packages we used. Given these limitations, we suggest that future research could evaluate longitudinal BODY-Q data to confirm the stability of the item calibrations both for the original Rasch-derived measures and for the bifactor IRT presented here.

Recently, a BODY-Q CAT was developed, which showed substantial item reduction of 37% for this comprehensive PROM [5]. The combination of a bifactor model with a multidimensional CAT might have the potential to establish an even more efficient and reliable BODY-Q CAT compared to this recently developed unidimensional CAT [13, 14].Supported by findings from the current study, further research is planned to investigate the performance and utility of a multidimensional CAT for the BODY-Q. Those interested in scoring using the bifactor model can use the parameters presented here in Table 3. Scoring is possible using the R Programming Environment and the mirt package. Our team is developing easy-to-use tools to facilitate online scoring which may be acquired by contacting the corresponding author.

The bifactor model proved to be a valuable tool for deriving overall appearance scores. Making use of a bifactor model for the BODY-Q adds value to the information gained from the PROM without increasing patient burden and without influencing regular BODY-Q items, responses, item parameters, or scoring. This method has the potential to further expand the utility of PROMs in clinical outcome assessment while mitigating the burden of response for patients.
