# Correction to: Deriving an overall appearance domain score by applying bifactor IRT analysis to the BODY‑Q appearance scales

**DOI:** 10.1007/s11136-020-02735-8

**Published:** 2021-03-20

**Authors:** Daan Geerards, Lisa van den Berg, Andrea L. Pusic, Maarten M. Hoogbergen, Anne F. Klassen, René R. W. J. van der Hulst, Chris J. Sidey‑Gibbons

**Affiliations:** 1 Patient‑Reported Outcomes, Value & Experience (PROVE) Center, Department of Surgery, Brigham and Women’s Hospital, 75 Francis St, Boston, MA 02115 USA; 2 Department of Surgery, Harvard Medical School, Boston, MA USA; 3 Department of Plastic and Reconstructive Surgery, Catharina Ziekenhuis, Eindhoven, The Netherlands; 4 Department of Pediatrics, McMaster University, Hamilton, ON Canada; 5 Department of Plastic and Reconstructive Surgery, Maastricht University Medical Center, Maastricht, The Netherlands; 6 Department of Symptom Research, University of Texas MD Anderson Cancer Center, Houston, TX USA

## Correction to: Quality of Life Research (2020) 29:1065–1072 10.1007/s11136-019-02366-8

In the original publication Table 1 has been removed as the authors did not obtain permission to reproduce the BODY-Q scale. A revised Table 1 is provided in this correction.

**Table 1:** Satisfaction with body scale

Item content^a^	Very dissatisfied	Somewhat dissatisfied	Somewhat satisfied	Very satisfied
1.. looks when dressed	1	2	3	4
2.. how clothes fit	1	2	3	4
3.. size	1	2	3	4
4.. Shape	1	2	3	4
5.. looks in photos	1	2	3	4
6.. looks from behind	1	2	3	4
7.. Looks from the side	1	2	3	4
8.. Looks in summer clothes	1	2	3	4
7.. Looks from the side	1	2	3	4
9.. Looks in a swimsuit	1	2	3	4
10.. Look in a mirror unclothed	1	2	3	4

^a^Item descriptions are not intended for replication. Please visit the Q Portfolio website for full item wording

The original article has been corrected.

